# A New Radiotranscriptomic Approach to Analyze Combined Sets of T3b Stage‐Specific Genes and Radiomic Features in Prostate Cancer

**DOI:** 10.1002/cnr2.70391

**Published:** 2025-12-12

**Authors:** Qian Yang, Peng Tang, Jiao Mo, Qiuyang Li, Jiahui Huang, Xiaoyu Han, Hao Xu, Xi Liu, Jie Tang

**Affiliations:** ^1^ Department of Ultrasound, Air Force Medical Center Air Force Military Medical University Beijing China; ^2^ Department of Ultrasound, First Medical Center Chinese PLA General Hospital Beijing China; ^3^ Department of Orthopedics, China Rehabilitation Research Center Beijing Charity Hospital Beijing China

**Keywords:** prostate cancer, radiomics, radiotranscriptomics, texture analysis, ultrasound

## Abstract

**Background:**

Current clinical staging of prostate cancer (PCa) using the tumor‐node‐metastasis (TNM) system and serum biomarkers remains limited in distinguishing locally advanced (T3b) PCa from organ‐confined (T2c) disease.

**Aims:**

Building on our previous biomarker discovery in differentiating PCa from that of benign prostatic hyperplasia, this study pioneers a radiotranscriptomic model to distinguish T3b stage PCa from T2c stage PCa by integrating contrast‐enhanced ultrasound (CEUS) radiomics with stage‐specific transcriptomic signatures, addressing a critical knowledge gap in precision staging.

**Methods and Results:**

This prospective study was approved by the review board of Chinese PLA General Hospital (S2021‐565‐01), and all participants provided written informed consent. Transrectal B‐mode ultrasound images and contrast‐enhanced ultrasound images on two imaging planes were prospectively analyzed in 48 patients with biopsy‐confirmed PCa (35 patients with stage T2c and 13 with stage T3b). Textural features were evaluated using microvascular ultrasonography and contrast‐enhanced ultrasound. Radiomic data were then retrieved from all modes. An across‐the‐board investigation of mRNA and miRNA expressions was also performed in the two PCa stages. Six biomarkers (frizzled 4, ribosomal protein S7, ribosomal protein L29, miR‐374c, miR‐9, and miR‐6510) were identified to differentiate T3b stage from T2c stage. The area under the curve (AUC) values of the combined set (AUC = 0.887, 0.956, and 0.996 for random forest, naïve Bayes, and support vector machine, respectively) and radiomic features alone (AUC = 0.921, 0.957, and 0.998, respectively) were found to be more accurate than those of the transcriptomic data alone (AUC = 0.583, 0.716, and 0.898, respectively) or clinical features alone (AUC = 0.585, 0.675, and 0.953, respectively). The PCa gene regulatory network comprised of four miRNAs (miR‐148, miR‐141, miR‐342, and miR‐210) may contribute to accelerating tumor progression.

**Conclusion:**

We established the new radiotranscriptomic signatures specifically optimized for differentiating T3b stage from T2c stage by decoding stage‐specific imaging‐genomic crosstalk. This new approach may overcome TNM staging limitations.

## 1 Introduction

Prostate cancer (PCa) represents the most prevalent malignancy in men worldwide, exhibiting remarkable interpatient heterogeneity and complex intratumoral molecular evolution that drives disease progression [1]. Current clinical staging through the tumor‐node‐metastasis (TNM) system and risk stratification using prostate‐specific antigen (PSA), Gleason grade groups, and clinical stage remain suboptimal for projecting pathological upstaging and metastatic potential [2, 3, 4]. Particularly, the critical distinction between organ‐confined (T2c) and locally advanced (T3b) disease carries major therapeutic implications, which, however, suffers from 30% to 40% discordance between clinical and pathological staging [5, 6].

Radiogenomic analysis has revealed that intratumor heterogeneity can be captured by imaging the entire tumor and the surrounding parenchyma. Recent advances in radiogenomic integration offer promising solutions to this classical diagnostic challenge. While conventional biomarkers focus on isolated molecular alterations, new radiotranscriptomic approaches enable spatial mapping of tumor biology by correlating multi‐parametric imaging features with transcriptomic profiles [7, 8]. While multiparametric magnetic resonance imaging (mpMRI) has emerged as the gold standard for tumor localization and risk stratification in Western clinical prostate oncology practice—offering superior soft‐tissue resolution and apparent diffusion coefficient (ADC) mapping for Gleason grade prediction [9]—its global accessibility remains constrained by cost, availability, and inter‐reader variability [10]. Emerging evidence suggests prostate ultrasound achieves comparable sensitivity (92% vs. 88%) and specificity (85% vs. 83%) to mpMRI in PCa detection while enabling real‐time targeted biopsies [11]. This modality's ability to visualize anterior lesions often missed by transrectal ultrasound (TRUS) biopsies [12], coupled with lower complication rates [13], positions it as a pragmatic alternative in resource‐limited settings. Contrast‐enhanced ultrasound (CEUS) includes the use of ultrasound contrast agents to visualize the vascular changes typical of prostate cancer, particularly angiogenesis, to assess cancer tissue perfusion and contrast diffusion [14].

Recent radiogenomic advances bridge imaging phenotypes with molecular heterogeneity. MRI radiomics leveraging ADC maps have successfully predicted Gleason grade groups by correlating diffusion restriction with cellular proliferation markers (e.g., Ki‐67) [15]. However, ultrasound‐based radiomics uniquely captures dynamic vascular signatures through CEUS, quantifying angiogenesis critical for PCa progression from indolent (< 2 mm) to clinically significant lesions [16]. Our prior work demonstrated that combining TRUS‐derived radiomics with plasma miRNA signatures improved PCa detection accuracy (AUC 0.998) compared to clinical datasets alone [17]. However, this model focused on distinguishing malignant pathology from benign lesions rather than staging discrimination, which is a critical knowledge gap addressed in the current study. Herein, we aimed to combine stage‐specific genes with imaging features associated with stage‐specific to construct a radiotranscriptomic signature to specifically target the T2c/T3b staging boundary where clinical decision‐making is most contentious. We also determined the relationship of newly identified candidate biomarkers, including frizzled 4 (FZD4), ribosomal protein S7 (PRS7), ribosomal protein L29 (RPL29), miR‐374c, miR‐6510, and miR‐9, with ultrasound (US) phenotype radiomic features, including textural and microvascular perfusion features.

## 2 Methods

### 2.1 Study Design and Cohort

Our previous paper reported new radiotranscriptomic differentiation between PCa and benign prostatic hyperplasia (BPH) [14]. This prospective study builds on our prior radiotranscriptomic cohort with a distinct analytical focus: stage‐specific discrimination between T2c (organ‐confined) and T3b (locally advanced) PCa. From November 2021 to May 2023, 22 healthy male controls and 48 patients with PCa (35 with stage T2c and 13 with stage T3b) underwent TRUS‐guided biopsies for PCa. We used multiple magnetic resonance imaging (MRI) scans to distinguish T2c stage from T3b stage. RNA‐sequencing was performed to analyze transcriptomic changes of mRNAs and miRNAs in the biopsy specimens.

### 2.2 Acquisition of US Images

We used an Esaote US system with a linear transducer (3–9 MHz, mechanical index: 0.04–0.13).

The details of the acquisition of US and CEUS images have been reported in our previous articles [17, 18]. The Medical Imaging Interactive Toolkit (MITK) was used to manually delineate the tumor boundaries and define the tumor region of interest (ROI) based on the texture feature of ultrasound image (B‐mode and CEUS images) types by a senior sonographer (> 10 years of work experience) [19]. We selected one frame for the analysis of texture features in CEUS images when the time–intensity curve (TIC) was at its peak time. The identified ROI lesions were then selected for biopsy.

### 2.3 Radiomic Textural Features

#### 2.3.1 Textural Feature Extraction

We defined 27 significant texture features in B‐mode images and 30 significant texture features in CEUS images to describe the tumor characteristics of the ROI in US images of stages T2c and T3b. Briefly, the texture analysis features included global, higher‐order texture, and wavelet features. Group 1 comprised six global features quantified using a first‐order histogram. Group 2 comprised 40 features. Group 3 comprised 322 wavelet‐filtered features. The wavelet features were named as follows: “cv1” and “cv2” are the vertical images, “ch1” and “ch2” were the horizontal images, and “cd1” and “cd2” were the 45° images of the decomposition of the first‐order and second‐order wavelets, respectively; “ca2” was the image sampled twice under wavelet decomposition.

### 2.4 Selection of Textural Features

A stepwise feature selection method was used to screen out the features that were most relevant to disease classification, but least relevant to other features. First, the feature most closely related to the disease classification variables was selected as the priming feature. Then, the feature most closely related to the disease classification variables but least related to the priming feature was selected as the second feature. The average of the correlation coefficients of this feature with all retained features, plus the correlation coefficients of this feature with the categorical variables, was used as the score for this feature. When the score of a feature dropped sharply, the stepwise feature selection procedure was terminated. Multivariate logistic regression, random forest, and a support vector machine (SVM) were used to train the disease classification models.

### 2.5 CEUS and Microvascular Perfusion Features

The microvascular perfusion features of CEUS images and video clips have been reported in our previous article [14].

### 2.6 Transcriptomic Analysis

The specific methods were presented in Appendix S1.

### 2.7 Construction of a PCa‐Specific Gene Regulatory Network

Our goal was to identify and combine transcriptional and post‐transcriptional regulatory processes between DEGs and DE miRNAs to illustrate this network regulatory key points in PCa between the two stages. The mRNAs that tended to continuously decrease in levels, and miRNAs that tended to continuously increase were selected and uploaded to the Disease‐specific miRNA/Transcription Factor Coregulatory Networks website for functional relationship prediction analysis.

### 2.8 Statistical Analyses

All data partitioning was conducted using R software. Data were analyzed using GraphPad Prism 9.0.2 and were summarized as the mean ± standard deviation (SD). Correlations between US imaging phenotypes and gene expression were analyzed using Pearson's correlation coefficients and the R package psych (Version 2.2.3). Unpaired two‐tailed Student's *t*‐tests or analysis of variance were used to compare two or multiple groups, respectively.

## 3 Results

### 3.1 Differentially Expressed Genes and Differentially Expressed MicroRNAs in PCa Patients Were Associated With Radiomic Features Related to T2c and T3b Stages

**FIGURE 1:**
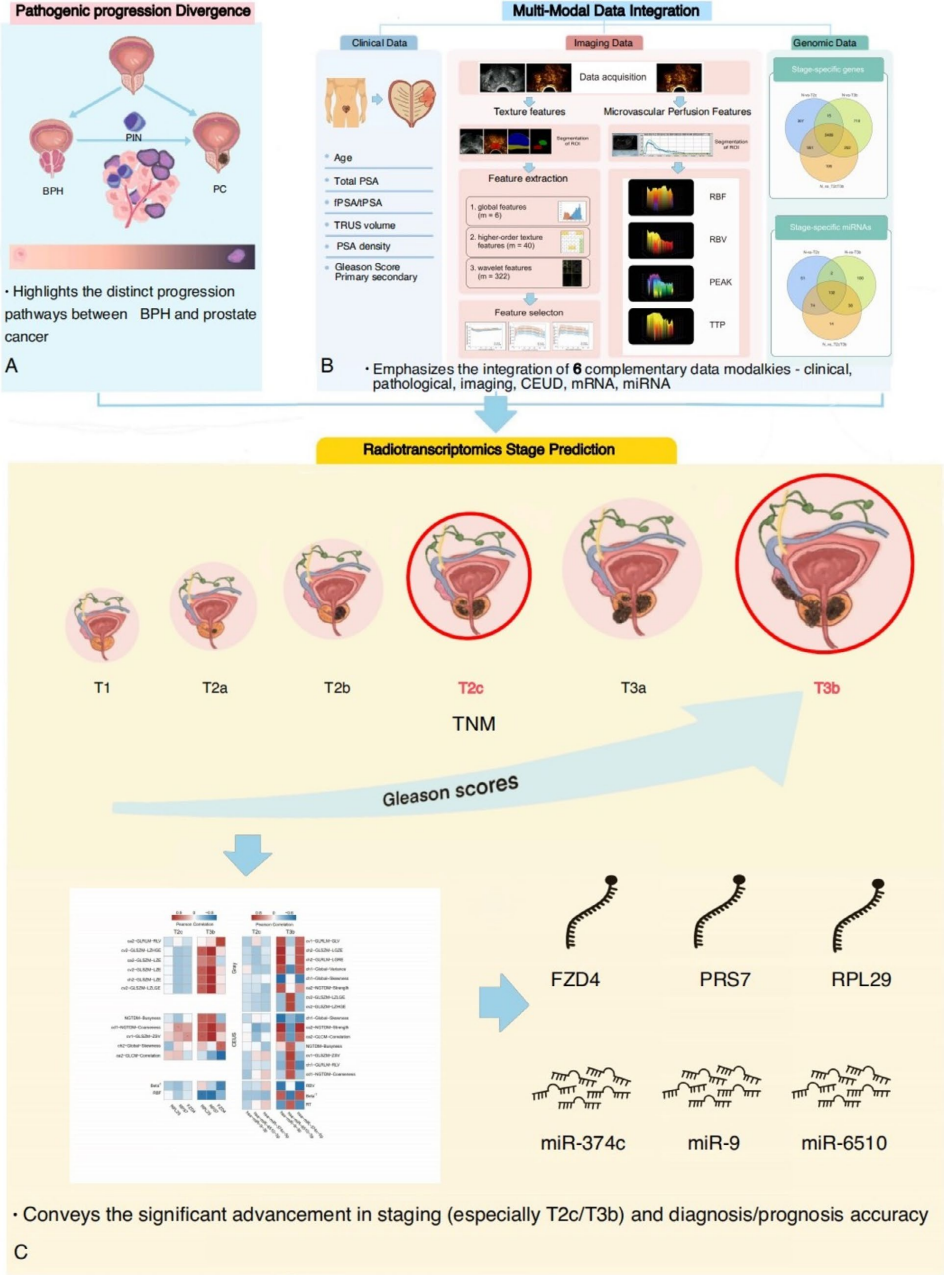
Illustration of the radiotranscriptomic workflow. (A) The pathogenic progression divergence: Highlights the distinct progression pathways between benign prostatic hyperplasia and prostate cancer. (B) Multi‐Modal Data Integration: Emphasizes the integration of 6 complementary data modalities—clinical, pathological, imaging, CEUS, mRNA, miRNA. (C) Radiotranscriptomic Stage Prediction: Conveys the significant advancement in staging (especially T2c/T3b) and diagnosis/prognosis accuracy.

To improve the precision of staging prediction, we applied radiomics to uncover the molecular process of PCa staging using clinical, imaging and transcriptomic data. The experimental design was shown in Figure 1. The pathogenic progression divergence was shown in Figure 1A. PCa progression staging is different from the pathways between benign prostatic hyperplasia (BPH) and prostate cancer. The multi‐modal data integration analysis was shown in Figure 1B. New radiotranscriptomic approaches have been developed in this study to integrate six major groups of data including: (1) clinical, (2) pathological findings, (3) ultrasound‐detected tissue image features, (4) CEUS:microvascular perfusion features; (5) Coding genomic transcriptomes—stage‐specific mRNAs; and (6) non‐coding genomic transcriptomes—stage‐specific microRNAs. By linking radiotranscriptomic stage prediction to identified biomarkers, we trained classifiers to predict pathological stage (Figure 1C). Our novel radiotranscriptomic findings provide better prostate cancer staging insights, especially for T2c and T3b, and superior diagnosis and prognosis compared to traditional clinical, pathological, ultrasound, or RNA analysis alone.

### 3.2 Analysis of Clinical Data in Patients With PCa Indicated That TRUS Volume in T2c Stage Was Significantly Lower Than That of T3b Stage

We collected clinical data and genes from 48 patients with PCa (T2c and T3b) and 22 healthy controls (Table 1) and found that TRUS volume in T2c stage was significantly lower than that of T3b stage.

**TABLE 1:** Clinical characteristics of the analyzed prostate cancer patient cohort.

Pathological stage	*N*	Gleason score primary + secondary	Age (y)	Total PSA (ng/mL)	fPSA/tPSA	TRUS volume (mL^3^)	PSA density (ng/mL^2^)
T2c	2	3 + 3	65.50 ± 7.78	11.60 ± 10.75	0.22 ± 0.12	61.00 ± 26.87	0.26 ± 0.29
	7	3 + 4	66.86 ± 5.90	14.94 ± 6.89	0.09 ± 0.02	28.00 ± 5.29	0.53 ± 0.19
	14	4 + 3	72.71 ± 8.98	16.41 ± 22.90	0.13 ± 0.07	43.07 ± 19.46	0.30 ± 0.21
	12	≥ 8	69.75 ± 7.33	57.36 ± 40.53*	0.10 ± 0.07	44.50 ± 22.67	0.72 ± 0.92
	35		70.11 ± 7.90	42.11 ± 33.66	0.12 ± 0.07	41.57 ± 20.03	0.49 ± 0.58
T3b	1	3 + 3	74.00 ± 0.00	28.40 ± 0.00	0.17 ± 0.00	33.43 ± 0.00	0.85 ± 0.00
	3	3 + 4	59.00 ± 2.65	28.33 ± 36.57	0.10 ± 0.11	60.67 ± 27.59	0.13 ± 0.03
	5	4 + 3	73.00 ± 6.82	27.69 ± 14.44	0.18 ± 0.14	41.80 ± 13.44	0.81 ± 0.66
	4	≥ 8	70.75 ± 0.00	73.80 ± 37.54*	0.12 ± 0.06	54.75 ± 21.97	0.32 ± 0.26
	13		68.46 ± 7.01	29.88 ± 33.92	0.11 ± 0.10	49.50 ± 19.86	0.50 ± 0.51

Abbreviations: fPSA, free PSA; PSA, prostate‐specific antigen; tPSA, total PSA.

* *p* < 0.05.

### 3.3 The Random Forest Model Was Used to Classify B‐Model Images, and the Support Vector Machine Model Was Used to Classify CEUS Images

For B‐mode images, the accuracy of the random forest model reached 0.89 out of 1.0 when using three features to construct the classification model. For CEUS images, the accuracy of the support vector machine (SVM) model was 0.84 out of 1.0 (Figure S1A–C).

### 3.4 T2c Was Enriched With the Pathways, Such as the Leukocyte Transendothelial Migration, Adherens Junction, and NF‐κB Signaling Pathway, While T3b Was Enriched With the Renin‐Angiotensin System, Hippo Signaling, and Cancer Pathways

**FIGURE 2:**
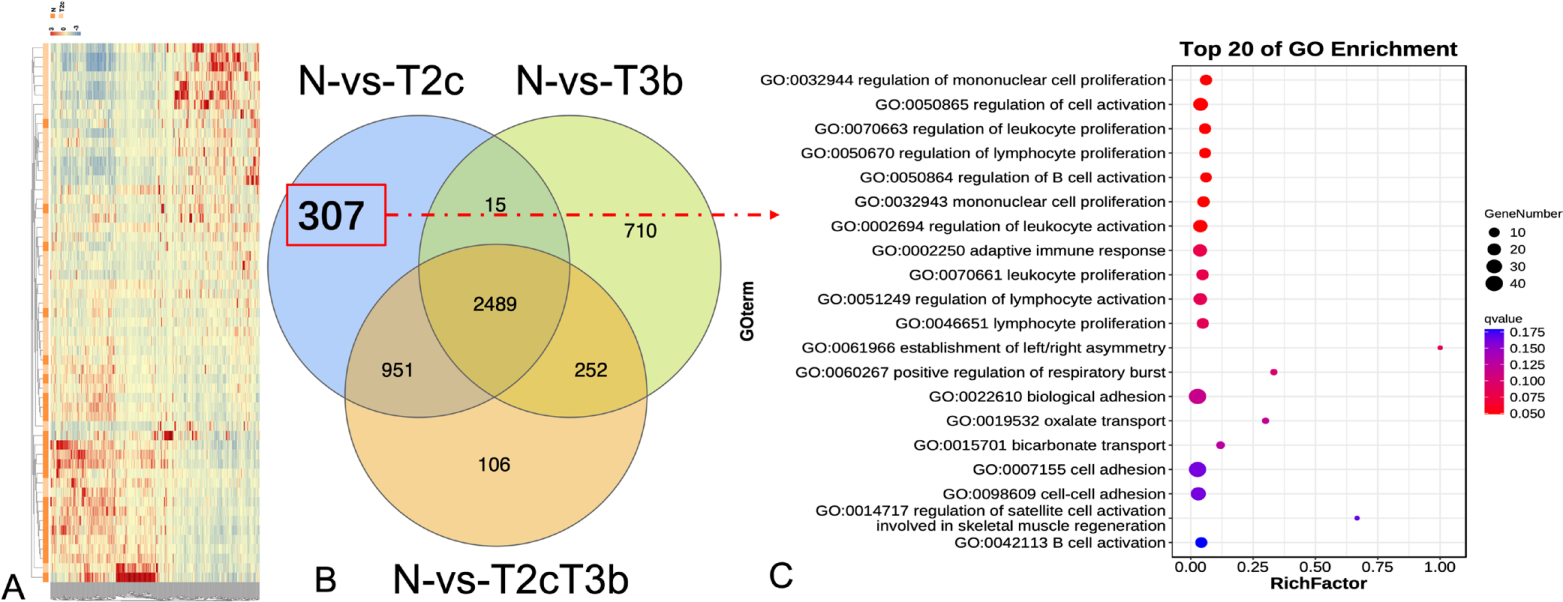
Functional characteristics of genes specific to stage T2c prostate cancer. (A) Heat map of the top 307 differentially expressed genes between stage T2c tumor samples and healthy samples. Blue denotes downregulation, whereas red denotes upregulation. (B) A Venn diagram of the overlap between the differentially expressed genes identified for stage T2c versus healthy samples, stage T3b versus healthy samples, and all tumor samples versus healthy samples. (C) Bubble chart showing the top 20 enriched Gene Ontology (GO) terms of biological processes for the 307 genes that are exclusively deregulated in the T2c tumor samples. The color and size of the bubble represent the enrichment significance and the number of differentially expressed genes enriched in GO terms, respectively. *Q*‐value < 0.05 was defined as significantly enriched.

**FIGURE 3:**
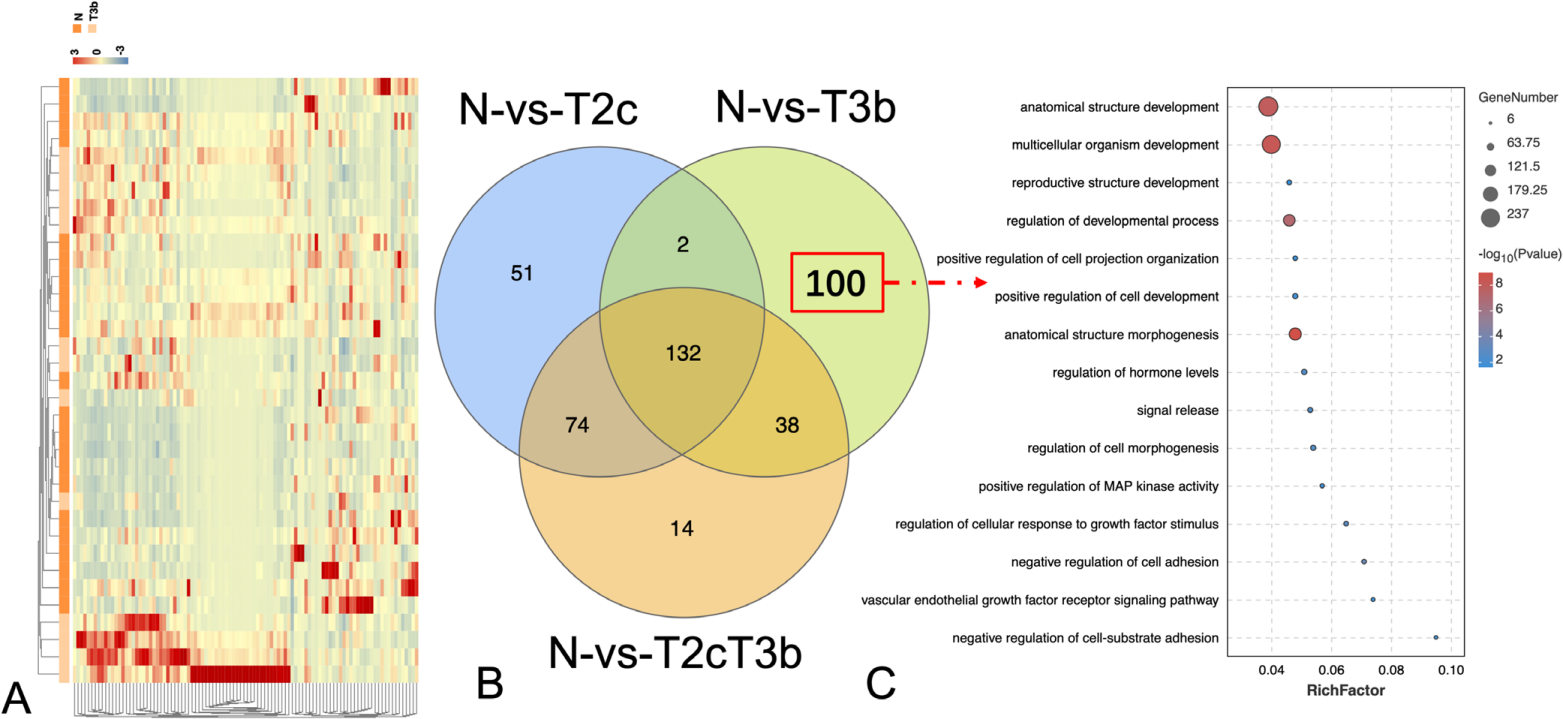
Functional characteristics of microRNAs (miRNAs) specific to stage T3b in prostate cancer. (A) The heat map for the top 100 differentially expressed miRNAs between stage T3b tumor samples and healthy samples. Blue denotes downregulation, whereas red denotes upregulation. (B) A Venn diagram of the overlap between the differentially expressed miRNAs identified for stage T2c versus healthy samples, stage T3b versus healthy samples, and all tumor samples versus healthy samples. (C) Bubble chart shows the enriched functional term specificity of the stage T3b miRNAs that are exclusively deregulated in the T3b tumor samples.

**FIGURE 4:**
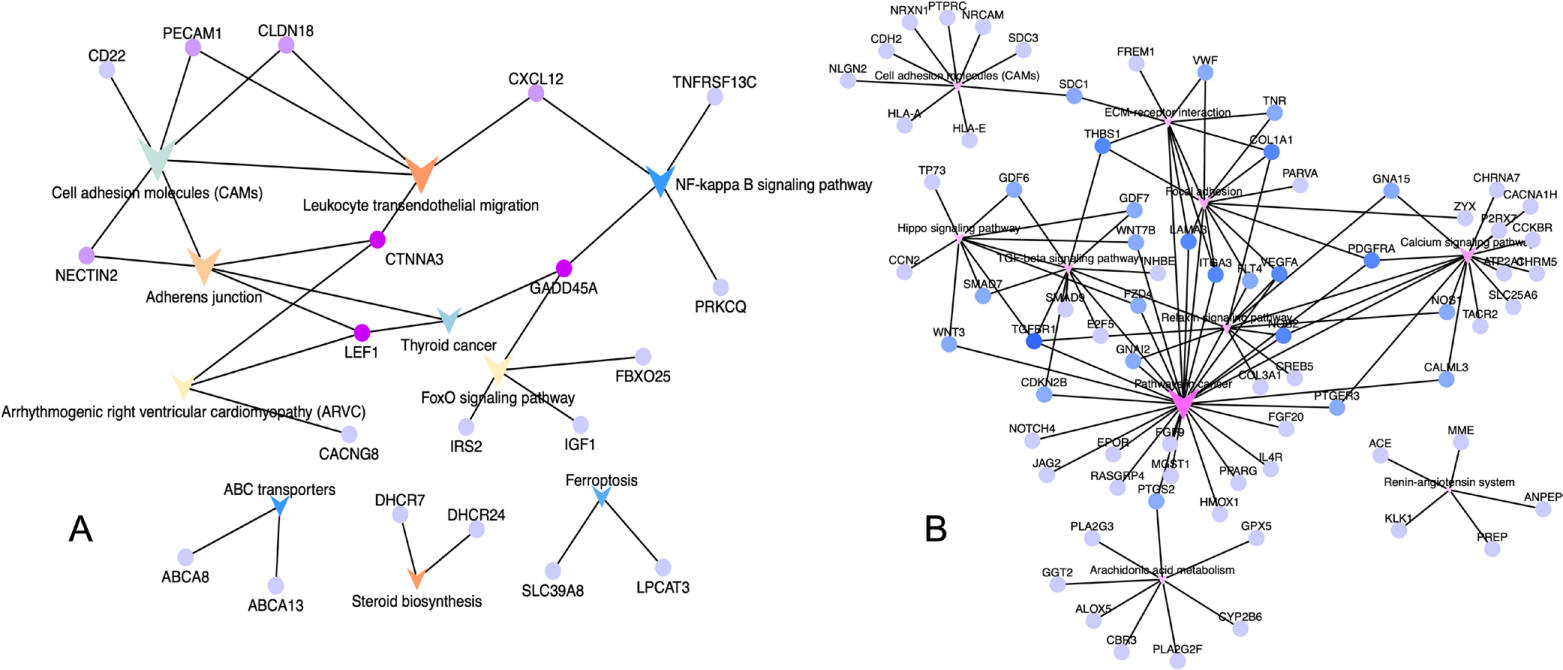
Potential biological functions in the two‐prostate cancer (PCa) stages: (A) Molecular network of pathways associated with stage T2c. (B) Molecular network of pathways associated with stage T3b.

In the stage‐specific differential expression analysis, we compared T2c and T3b tissues with healthy tissues and selected DEGs and DE miRNAs that were specific to each stage (i.e., that were not identified as T2c/T3b‐specific DEGs or DE miRNAs in comparisons between the other stage and healthy tissues or between all healthy tissues). The results revealed 307 stage‐specific DEGs and 51 stage‐specific DE miRNAs for stage T2c (Figures 2A–C and S2). In addition, 710 stage‐specific DEGs and 100 stage‐specific DE miRNAs were identified for stage T3b (Figures 3 and S3). Functional analyses of these stage T2c‐, and T3b‐, specific DEGs and DE miRNAs revealed different functional GO terms and KEGG pathways. The enriched functions for T2c‐specific DEGs included directive mononuclear cell and lymphocyte proliferation as well as the regulation of cell adhesion and cell activation, which were known to be related to the occurrence of prostate tumors and were consistent with stage T2c. The top 20 most important GO terms were shown in Figure 2C. The T3b‐specific DEGs were associated with a range of GO terms (Figure S3C). Multicellular organism development, anatomical structural development, regulation of developmental processes, and cellular secretion were found in both primary and metastatic PCa, which were consistent with stage T3b and associated with increased PCa risk. Similar analyses were performed for the DE miRNAs at each stage (Figure 3). We listed the important KEGG pathways enriched in T2c and T3b stage‐specific genes that could be related to PCa stages (Tables 2 and 3). The pathway enrichment analysis showed that T2c was enriched with the pathways, such as the leukocyte transendothelial migration, adherens junction, and NF‐κB signaling pathway, while T3b was enriched with the renin–angiotensin system, hippo signaling, and cancer pathways as we reported [20, 21, 22]. Figure 4 showed the molecular network of the relevant pathways with two PCa stages: T2c and T3b.

**TABLE 2:** Enriched pathways which characterize the T2c stage but not the T2c stage.

Pathways	Involved genes	*p*
Leukocyte transendothelial migration	CXCL12; CTNNA3; CLDN18; PECAM1	0.01
Steroid biosynthesis	DHCR24; DHCR7	0.01
Adherens junction	CTNNA3; LEF1; NECTIN2	0.01
FoxO signaling pathway	IRS2; FBXO25; IGF1; GADD45A	0.02
Arrhythmogenic right ventricular cardiomyopathy (ARVC)	CTNNA3; LEF1; CACNG8	0.02
Cell adhesion molecules (CAMs)	CD22; NECTIN2; CLDN18; PECAM1	0.03
Thyroid cancer	LEF1; GADD45A	0.03
Ferroptosis	LPCAT3; SLC39A8	0.04
NF‐kappa B signaling pathway	TNFRSF13C; PRKCQ; CXCL12; GADD45A	0.04
ABC transporters	ABCA13; ABCA8	0.04

**TABLE 3:** Enriched pathways which characterize the T3b stage but not the T3b stage.

Pathways	Involved genes	*p*
Renin‐angiotensin system	ANPEP; PREP; KLK1; MME; ACE	< 0.01
Arachidonic acid metabolism	CBR3; GGT2; PLA2G3; ALOX5; GPX5; PLA2G2F; CYP2B6; PTGS2	< 0.01
TGF‐beta signaling pathway	SMAD9; SMAD7; CDKN2B; THBS1; TGFBR1; E2F5; GDF6; GDF7; INHBE	< 0.01
ECM‐receptor interaction	LAMA3; FREM1; THBS1; VWF; SDC1; TNR; ITGA3; COL1A1	0.01
Relaxin signaling pathway	CREB5; TGFBR1; GNA15; VEGFA; GNAI2; NOS1; COL3A1; NOS2; COL1A1	0.02
Pathways in cancer	FGF9; LAMA3; HMOX1; FGF20; WNT7B; CDKN2B; CALML3; TGFBR1; PDGFRA; NOTCH4; MGST1; IL4R; EPOR; PPARG; VEGFA; JAG2; RASGRP4; GNAI2; FLT4; PTGER3; FZD4; PTGS2; NOS2; WNT3; ITGA3	0.03
Calcium signaling pathway	CALML3; SLC25A6; CHRNA7; CHRM5; CACNA1H; GNA15; PDGFRA; TACR2; ATP2A1; CCKBR; NOS1; P2RX7; PTGER3; NOS2	0.04
Cell adhesion molecules (CAMs)	CDH2; HLA‐A; HLA‐E; SDC1; NRCAM; NRXN1; SDC3; PTPRC; NLGN2	0.04
Focal adhesion	LAMA3; THBS1; PDGFRA (VWF); VEGFA; PARVA; ZYX; FLT4; TNR; ITGA3; COL1A1	0.04
Hippo signaling pathway	SMAD7; WNT7B; TGFBR1; GDF6; GDF7; TP73; CCN2; FZD4; WNT3	0.04

### 3.5 Six Textural Features Highly Correlated With DEGs, and Eight Textural Features Highly Correlated With DE miRNAs Were Identified for the B‐Mode US; and Five Textural Features and Two Microvascular Perfusion Features Highly Correlated With DEGs and Seven Textural Features and Three Microvascular Perfusion Features Highly Correlated With DE miRNAs Were Identified for the CEUS

To interrogate the association of expression and radiomic textural features, we used pie‐chart mapping to understand all pairwise correlations between significant textural features, microvascular perfusion, and stage T2c and T3b DEGs and DE miRNAs. According to the B‐mode US, six significant textural features that were highly correlated with DEGs were selected: ca2−GLRLM−RLV, cv2−GLSZM−LZHGE, ca2−GLSZM−LZE, cv2−GLSZM−LZE, ch2−GLSZM−LZE, and cv2−GLSZM−large zone low gray‐level emphasis (LZLGE). Eight significant textural features that were highly correlated with DE miRNAs were also selected, including cv1−GLRLM−gray level variance, ch2−GLSZM−LGZE, ch2−GLRLM−LGRE, ch1−Global−Variance, ch1−Global−Skewness, ca2−NGTDM−Strength, cv2−GLSZM−LZLGE, and v2−GLSZM−LZHGE.

**FIGURE 5:**
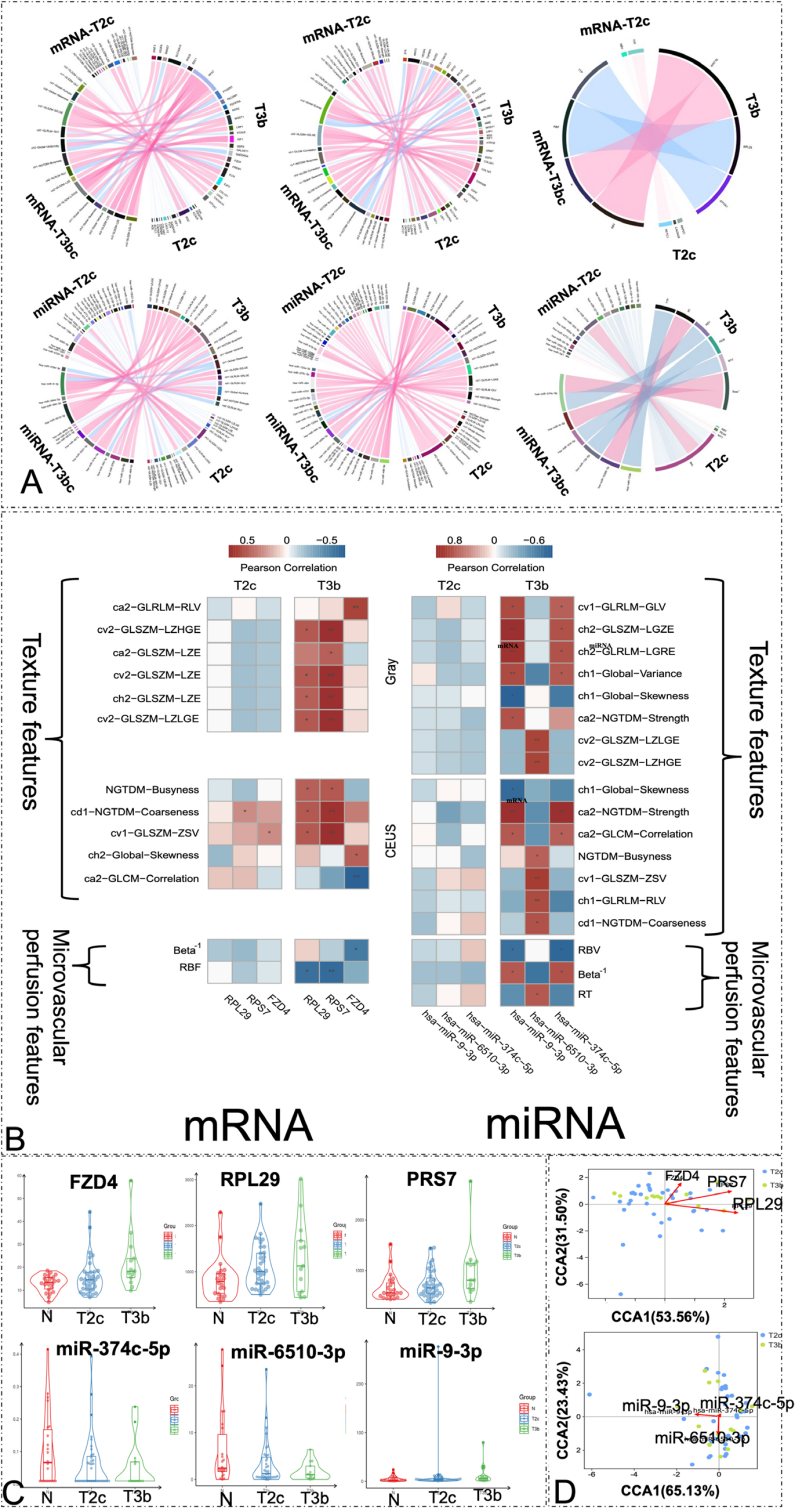
Summary of radiotranscriptomic correlations. (A‐i) Colored circles refer to 27 significant textural features of B‐mode ultrasound and genes; (A‐ii) colored circles refer to 30 significant textural features of contrast‐enhanced ultrasound (CEUS) and genes; (A‐iii) colored circles refer to four significant microvascular perfusion features of CEUS imaging and genes; (A‐iv) colored circles refer to 27 significant textural features of B‐mode ultrasound and microRNAs (miRNAs); (A‐v) the colored circles refer to 30 significant textural features of CEUS and miRNAs; (A‐vi) the colored circles refer to four significant microvascular perfusion features of CEUS imaging and miRNAs. The left half of the pie chart represents the radiomic features, and the right half represents related genes. Ribbons connecting textural features and genes refer to significant correlations between them. Ribbon color refers to the correlation coefficient sign (red, positive; blue, negative). (B) This section shows the correlation matrix between the expression levels of the six biomarkers and the extracted stage‐specific radiomic textural features and microvascular perfusion features. The significant correlations (*p* < 0.05) are marked with an asterisk (*); *p* < 0.01 is marked with two asterisks (**). (C) The expression levels of the six molecular (“genomic”) biomarkers in tumor stages T2c and T3b. (D) Canonical correspondence analysis of the distribution influence for these six biomarkers revealed reasonable separation between the T2c and T3b samples. The cosine value of the angle between the arrow and the ranking axis represents the correlation between characteristic genes and the ranking axis. mRNA, messenger RNA.

According to the CEUS, five significant textural features (NGTDM−Busyness, cd1−NGTDM−Coarseness, cv1−LZLGE−ZSV, ch2−Global−Skewness, and ca2−GLCM−correlation) and two microvascular perfusion features (beta^−1^ and regional blood flow) that were highly correlated with DEGs were selected. Moreover, seven significant textural features (ch1−Global−Skewness, ca2−NGTDM−Strength, ca2−GLCM−Correlation, NGTDM−Busyness, cv1−GLSZM−ZSV, ch1−GLRLM−RLV, and cd1−NGTDM−Coarseness) and three microvascular perfusion features (RBV, beta^−1^ and RT) that were highly correlated with DE miRNAs were selected (Figure 5A).

### 3.6 Upregulations of Four Genes Were Identified as T3b Stage‐Specific Biomarkers, Which Were Well Correlated With Stage‐Specific US Imaging Features at T2c and T3b

We examined PCa biomarkers association with the stages‐related imaging features identified from the consistent US phenotypes of men with PCa. We calculated the correlation between the biomarkers and US imaging features at the two stages (T2c and T3b). For patients with stage T3b, the strongest positive correlations were noted between ribosomal protein subunit 7 (RPS7) expression data (max *r* = 0.89, *p* < 0.05) and the significant textural features (cv2–GLSZM–LZE and cv2–GLSZM–LZLGE) associated with B‐mode US as well as between RPS7 expression data (max *r* = 0.89, *p* < 0.05) and the significant textural features (cv1–GLSZM–ZSV) associated with CEUS (Figure 5B). For patients with stage T3b, the strongest positive correlations were between miR‐9‐3p expression data (max *r* = 0.83, *p* < 0.05) and the significant textural features (ch2–GLSZM–LGZE) associated with B‐mode US as well as between miR‐9‐3p expression data (max *r* = 0.85, *p* < 0.05) and the significant textural features (ca2–NGTDM–Strength) associated with CEUS (Figure 5B). Microvascular perfusion characteristics can also be used as powerful indicators to distinguish between T2c and T3b PCa and provide insights into their underlying biology. The strongest negative correlation was between RPS7 expression (max *r* = −0.69, *p* < 0.05) and the significant microvascular perfusion features (regional blood flow, RBF) associated with CEUS, while the strongest positive correlations were between has‐miR‐6510‐3p expression data (max *r* = −0.63, *p* < 0.05) and the significant microvascular perfusion features (refilling time, RT) associated with CEUS in patients with stage T3b PCa. Notably, patients with stage T2c and T3b PCa showed significant differences in imaging characteristics (Figure 5B and Tables S1 and S2). Further, we identified the DEGs FZD4, RPS7, and RPL29 and the DE miRNAs miR‐374c, miR‐6510, and miR‐9‐3p using a *p*‐value cutoff of 0.05 and an increased fold change threshold of 1.5. FZD4, RPS7, RPL29, and miR‐9‐3p were upregulated in the T3b stage, whereas miR‐374c and miR‐6510 were downregulated in the T3b stage (Figure 5C). Canonical correspondence analysis of the effects of the distribution of these six biomarkers revealed significant deviations between the two stages (Figure 5D).

### 3.7 The Combined Set of Three Groups of Data Such as Clinical Data, Transcriptomic Biomarkers, and Radiomic Features Showed a More Accurate Power Than Each Set Alone for Accurate PCa Prognosis

**FIGURE 6:**
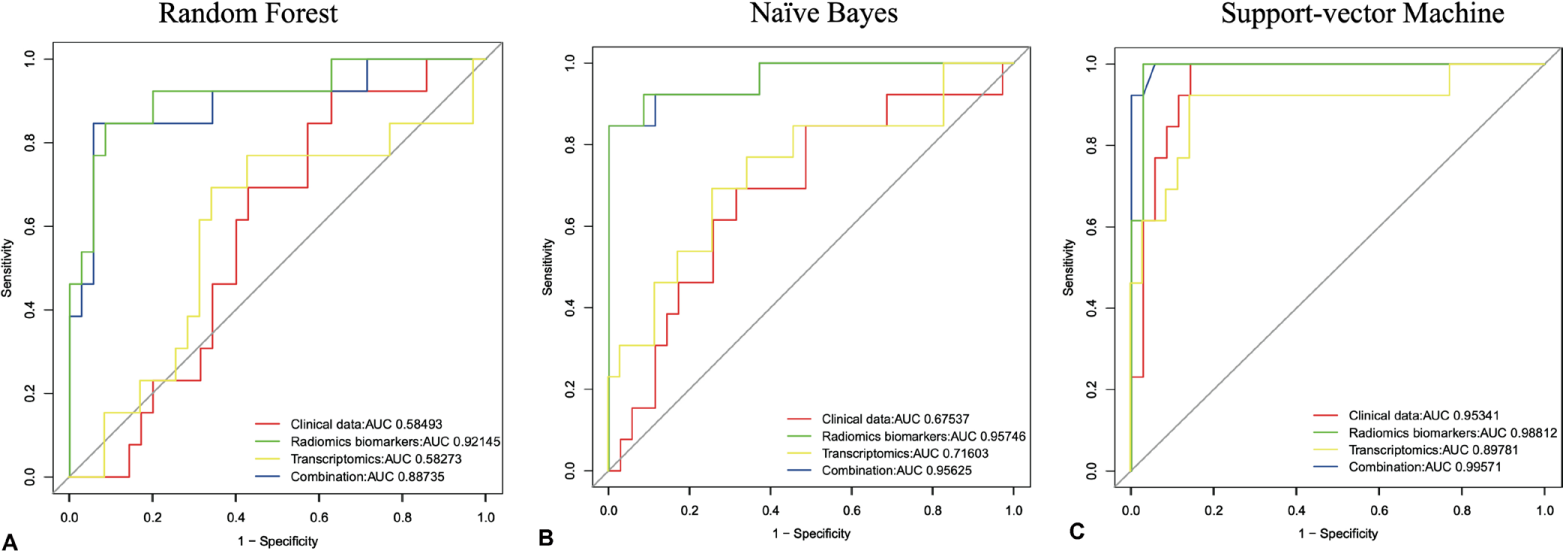
Prediction of performance using the clinical features, transcriptomic features, radiomic features, and combined features for predicting the pathological stage (T2c vs. T3b). The shown receiver operating characteristic curves are representative of the receiver operating characteristic curves obtained from the three prediction methods, selected because their area under the curve value is closest to the average area under the curve value of the respective methods over all 10 runs (see also Table S3).

We used three machine‐learning methods and receiver operating characteristic curves to evaluate the prediction accuracy and dependability of all the features in four feature sets: the (1) clinical data set; (2) transcriptomics biomarkers (FZD4, RPS7, RPL29, miR‐374c‐5p, miR‐9‐3p, and miR‐6510‐3p); (3) radiomic features (textural and microvascular perfusion); (4) combined set. The combined set (AUC = 0.887, 0.956, and 0.996 for random forest, naïve Bayes, and SVM, respectively) and radiomic features (AUC = 0.921, 0.957, and 0.998, respectively) showed a more accurate prognostic power than the clinical dataset (AUC = 0.585, 0.675, and 0.953, respectively) or transcriptomic (gene and miRNA) features (AUC = 0.583, 0.716, and 0.898, respectively) (Table S3). These results emphasize the possibility of combining features from various datasets to obtain more accurate prognoses for patients with PCa (Figure 6).

### 3.8 A New PCa Gene Regulatory Network With Four Central miRNAs That Regulated Nine Target Genes Was Identified

**FIGURE 7:**
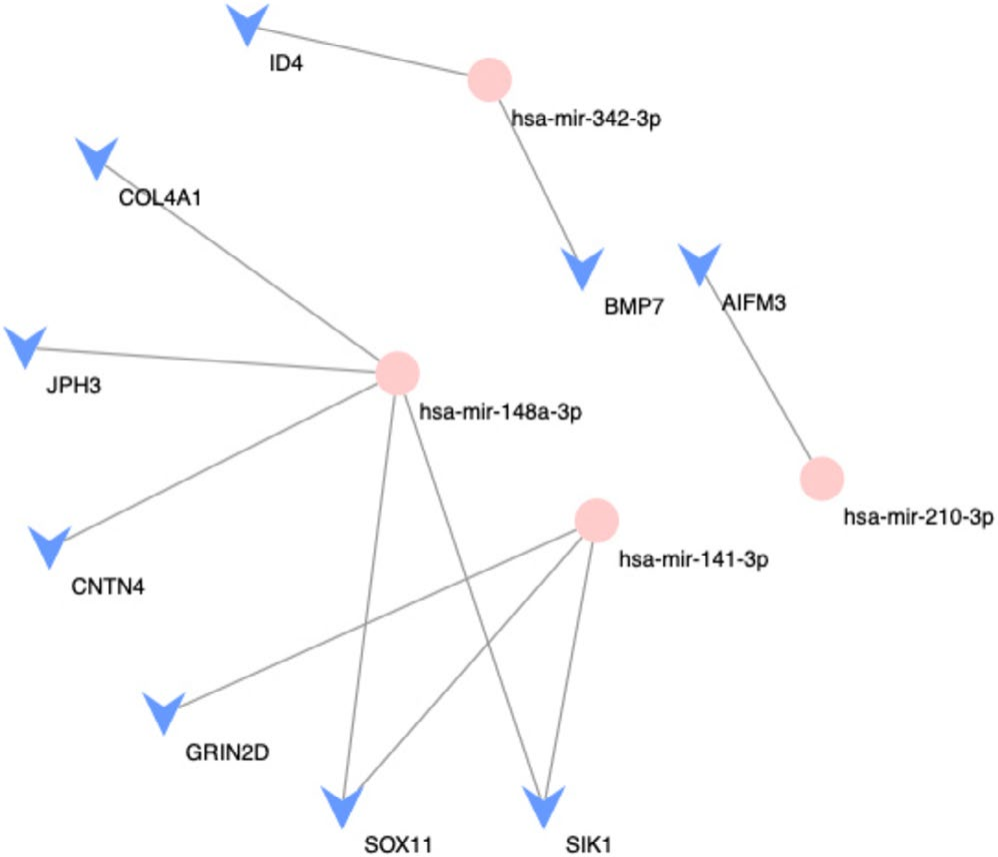
Prostate cancer gene regulatory network constructed from the microRNAs and genes that were differentially expressed between the T2c and T3b tumor samples. The pink circles represent potential driver microRNAs, whereas the blue arrow heads represent genes.

To determine the influence of these miRNAs and describe the key points that contribute significantly to the general rule, we assessed the importance of key point factors, performed stage‐specific analyses of key points according to their degree of importance, and identified network hubs. We identified four central miRNAs (miR‐148, miR‐141, miR‐342, and miR‐210) that regulated nine target genes in the PCa–GRN network, maintaining the connections between these miRNAs and their adjoining genes (Figure 7).

## 4 Discussion

While textural analysis of TRUS images has been established in our previous report for distinguishing benign from malignant prostate tissues [23, 24], our study pioneers contrast‐enhanced ultrasound (CEUS) radiomics integration with stage‐specific gene expression. Critically, this addresses a fundamental gap in radiotranscriptomic research: as summarized in Table 4, recent radiotranscriptomic advances demonstrate distinct modality‐specific strengths: multiparametric MRI (mpMRI) excels in correlating perfusion features with key transcriptional regulators like STAT6/TRAP2A [25], while PSMA‐PET/CT achieves robust metastasis prediction through maximum standardized uptake value (SUVmax) quantification (AUC = 0.89) [26]. Though ultrasound has shown preliminary correlations with general PCa biomarkers [17], our work uniquely addresses the critical gap in stage‐defined biomarker discovery.

**TABLE 4:** Radiometric and radiotranscriptomic reports in the prostate cancer research field—(such as MRI, PSMA‐PET/CT and ultrasound), combining either with transcriptomic or with prostate‐specific membrane antigen detection, have been developed to improve diagnosis, therapeutics and prognosis of prostate cancer—a comparison.

Radiometric and radiotranscriptomic reports in the prostate cancer research field—three different imaging technologies	Radiometrics or radiotranscriptomics	Methods	Sample sizes	Key findings	Journal/Year/PMID
Radiogenomics analysis linking multiparametric magnetic resonance imaging MRI and transcriptomics	Radiotranscriptomics	Correlation of multiparametric MRI texture features (T2W/DWI/DCE) and pharmacokinetic parameters with transcriptomic features (transcription factors/pathway activity) from RNA‐seq.	A final set of 31 imaging features was correlated to 33 transcriptomic features obtained on the same tumors.	Significant inverse correlations between perfusion feature (MRDIA median) and STAT6/TRAP2A activities (*r* = −0.64/−0.50). T2W texture features associated with STAT6‐mediated tumor proliferation/migration.	Cancers, 2023; PMID: 37370685
Predicting the Risk of Metastases by imaging using prostate‐specific membrane antigen‐positron emission tomography/computed tomography (PSMA‐PET/CT)	Radiometrics	Metastasis prediction model using intraprostatic maximum standardized uptake value (SUVmax) quantification and multivariable logistic regression with receiver operating characteristic (ROC) curves analysis.	335 men with biopsy‐proven prostate carcinoma and imaging using prostate‐specific membrane antigen‐positron emission tomography/computed tomography (PSMA‐PET/CT) for primary staging were enrolled in the present, retrospective study.	Intraprostatic SUVmax identified as independent metastasis predictor. Combined model (SUVmax + ISUP grade) achieved AUC = 0.89 for metastatic risk stratification.	Cancers, 2021; PMID: 33805971
Radiotranscriptomics with ultrasound image analysis (A previous report from the authors of current study)	Radiotranscriptomics	Integration of B‐mode ultrasound texture features, CEUS microvascular perfusion parameters, and RNA‐seq data for prognostic modeling.	Radiotranscriptomic analyses were performed of clinical, imaging, and two genomic (mRNA and microRNA expression) datasets from 48 and 22 men with PCa and benign prostatic hyperplasia (BPH), respectively.	23 US/CEUS features correlated with 52 differentially expressed genes (*p* < 0.05). CEUS parameters reflected genomic alterations in hormone receptors and prognostic biomarkers.	Cancer Medicine, 2023; PMID: 37987209

Among the different texture analysis methods, wavelet transformation and time–frequency analysis techniques stand out in terms of algorithmic efficiency [27, 28]. In our research, the 322 wavelet features were the intensity‐, and texture‐features of the corresponding PCa ROIs in the wavelet‐decomposed images. We found that the wavelet‐derived features (e.g., ch1–Global–Skewness, ca2–NGTDM–Strength) identified in T3b tumors reflect distinct vascular heterogeneity patterns. Unlike Sun et al.'s horizontal GLCM–GLRLM comparisons for “aggressiveness” classification [29], our CEUS radiomics pipeline has prioritized stage‐specific irregularity detection through wavelet decompositions (e.g., cv2–GLSZM–LZHGE), while NGTDM–Busyness, cv1–GLSZM–ZSV, and ca2–GLCM–Coarseness were recurring important textural features of CEUS, both in patients with stage T2 and T3b. This approach achieved superior accuracy. Critically, these features were absent in our prior benign/malignant model, underscoring their specificity for PCa stage discrimination rather than malignancy detection.

Our detailed RNA‐seq data analyses of DEGs and DE miRNAs based on clinical and histopathological features highlighted the considerable synergistical integration between US imaging and transcriptomics in PCa. Our results demonstrated that gene expression can be used to identify at‐risk patients with PCa. RPS7 promotes cell migration by regulating the epithelial–mesenchymal transition in PCa [30]. FZD4 is a moderator of oncogene‐induced signaling, and the epithelial–mesenchymal transition is significantly upregulated in PCa cells, driving PCa bone metastatic tropism and invasion [31, 32, 33, 34, 35]. The downregulation of RPL29 can reduce the proliferation and invasion of squamous cell carcinomas [36]. CEUS enables the visualization of prostate areas with abnormal vascularity, which can improve the number of positive biopsies performed [37]. Microvascular perfusion characteristics can also act as strong indicators of PCa and provide insights into its underlying biology. The association between CEUS refilling time and DEGs provides mechanistic insights into T3b vascular remodeling. These findings extend beyond microvascular perfusion correlations by linking imaging phenotypes to stage‐specific molecular drivers—a critical step toward personalized therapy stratification.

miRNAs are small non‐coding RNA molecules involved in gene expression regulation at the post‐transcriptional level. They target PCa‐associated mRNAs and may promote or inhibit PCa metastasis. They also represent potential biomarkers of therapy responses and metastasis, subject to preclinical evaluation, in addition to their potential as therapeutic targets [38, 39]. Our results support the notion that gene and miRNA expression can be utilized for the improved clinical management of at‐risk patients [40]. The roles of miR‐374c‐5p as a potential therapeutic target in PCa [41, 42, 43] or as a tool to suppress PCa cell viability, invasion, migration, proliferation, and promote apoptosis have been reported [44]. Certain miRNAs, such as miR‐9‐3p, increase the metastatic potential of PCa cells [45, 46]. Abnormal miR‐6510 expression is involved in tumorigenesis and the progression of various malignancies [47]. Taken together, these miRNAs, when integrated with textural features, provide a PCa stage‐specific diagnostic framework rather than generic “aggressiveness” classifiers.

Current developments in the collection and analysis of medical images allow for the high‐throughput filtering of data features to accurately calculate changes in tumor tissues [48]. The identified imaging biomarkers included genes and miRNAs reflecting PCa characteristics and were highly associated with invasive radiomic features obtained from US images. When combined with clinical data and machine learning, these biomarkers can be used to improve the estimation of PCa stages. For patients with stage T2c PCa, the textural features were significantly more reliable than microvascular perfusion features in relation to biomarker expressions. For patients with stage T3b disease, the correlation between radiomic features and biomarkers was stronger than that in the group with stage T2 disease. This result is consistent with the reasonable diagnostic accuracy of radiomic features, particularly in patients with progressive PCa. This significantly improved the capacity for PCa stage prediction. Consistent with our outcomes, Shen et al. also distinguished high‐risk and low‐risk patients with aggressive PCa using miR‐20a, miR‐21, miR‐145, and miR‐221, with an AUC of 0.801 [49]. Recent studies have also used other machine learning models, such as neurofuzzy models and artificial networks [50, 51] to predict PCa stage based solely on clinical characteristics. They reported AUC values (0.812 and 0.695, respectively) that were similar to those calculated by our machine learning methods using clinical characteristics. Remarkably, in all three methods for predicting pathological stages, predictions based on the combined dataset had the largest AUC (AUC = 0.887, 0.956, and 0.996 for random forest, naïve Bayes, and SVM, respectively) and were superior to the prediction methods based on the clinical, radiomic, and transcriptomic feature datasets alone, respectively.

Many studies have identified miRNAs as promoters or inhibitors of metastasis and have partially uncovered their regulatory pathways in PCa. Our PCa–GRN showed that miRNAs (miR‐148, miR‐141, miR‐342, and miR‐210) play key roles in PCa development from T2c to T3b, providing novel mechanistic insights into localized progression from T2c (organ‐confined) to T3b (extracapsular) disease. While miR‐148‐3p is recognized as a metastasis suppressor [52, 53, 54], its *stage‐specific* downregulation in T3b tumors (*p* < 0.001) coincides with Wnt/β‐catenin‐driven stromal invasion [52, 53, 54, 55, 56, 57]. The abnormal expression patterns of candidate driver metastatic miRNAs, such as miR‐210‐3p and miR‐342‐3p, increase the potential of PCa cells to metastasize [58]. Our findings highlight the advances prior miRNA studies focused on metastatic potential by anchoring molecular drivers to stage‐defined imaging phenotypes, significantly addressing TNM staging discordance.

Our study has three key limitations: First, the T3b subgroup size (*n* = 13) and single‐center design may introduce selection bias, necessitating multi‐ethnic validation in larger cohorts like the PROSTATEx‐2024 dataset [59]. Second, reliance on biopsy specimens rather than whole‐mount prostatectomy samples could underrepresent tumor heterogeneity—a concern partially mitigated by CEUS whole‐gland analysis. Most critically, while our cross‐sectional design robustly identifies T3b‐associated extracapsular extension, it cannot prognosticate metastatic evolution in clinically localized T2c tumors—a fundamental limitation of current TNM staging. Future longitudinal validation must evaluate whether mRNA/miRNA panels—CEUS vascular heterogeneity predict metastasis‐free survival, addressing the urgent clinical imperative to distinguish indolent T2c lesions from those harboring lethal metastatic potential.

In conclusion, this study establishes the first radiotranscriptomic framework for stage‐specific PCa discrimination. The results of our transcriptomic analysis clearly demonstrated that using machine learning to analyze combined sets of clinical data, T3b stage‐specific genes, and radiomic features has exhibited greater prognostic power for patients with PCa than traditional approaches.

## Author Contributions

**Qian Yang:** conceptualization; data curation; formal analysis; funding acquisition; investigation; methodology; resources; writing – original draft; writing – review and editing. **Peng Tang:** conceptualization; resources. **Jiao Mo:** data curation; software. **Qiuyang Li:** data curation; formal analysis; validation. **Jiahui Huang:** data curation; software; validation. **Xiaoyu Han:** formal analysis; investigation; software. **Hao Xu:** data curation; resources. **Xi Liu:** project administration; supervision. **Jie Tang:** supervision.

## Funding

This work was supported by National Social Science Fund of China, 2024‐SKJJ‐B‐047; National Key Research and Development Program of China, 2024YFB3214400; Natural Science Basic Research Program of Shaanxi Province, 2023‐JC‐QN‐0912.

## Consent

The requirement for patient consent was waived by the ethics committees owing to the retrospective nature of the study.

## Conflicts of Interest

The authors declare no conflicts of interest.

## Supporting information


**Figure S1:** Evaluation models of prostate cancer based on radiomics features. (A) LR = logistic regression mode. (B) SVM = support vector machine mode. (C) RF = random forest mode.
**Figure S2:** Functional characteristics of the miRNAs specific to stage T2c in PCa. (A) The heat map for the top 51 differentially expressed (DE) miRNAs between T2c tumor samples and healthy samples. Blue denotes down‐regulation whereas red denotes up‐regulation. The dendrograms on the upper and left sides show the hierarchical clustering tree of samples and miRNAs, respectively. (B) A Venn diagram of the overlap between the DE miRNAs identified for T2c versus healthy samples, stage T3b versus healthy samples, and all tumor samples versus healthy samples. (C) Bubble chart shows the enriched functional terms specificity of the T2c miRNAs that are exclusively deregulated in the T2c tumor samples.
**Figure S3:** Functional characteristics of genes specific to stage T3b in PCa. (A) The heat map for the top 710 differentially expressed genes (DEG) between T3b tumor samples and healthy samples. Blue denotes down‐regulation whereas red denotes up‐regulation. The dendrograms on the upper and left sides show the hierarchical clustering tree of samples and genes, respectively. (B) A Venn diagram of the overlap between the DEGs identified for T2c versus healthy samples, stage T3b versus healthy samples, and all tumor samples versus healthy samples. (C) Bubble chart shows the top 20 enriched Gene Ontology (GO) terms of biological processes of 710 genes that are exclusively deregulated in the T3b tumor samples, based on the GO semantic similarities. Color and size of the bubble represented enrichment significance and the number of DEGs enriched in GO terms, respectively. Q‐value < 0.05 was defined as significantly enriched.
**Table S1:** Differentially expressed genes relevant to prostate cancer according to the ultrasound phenotype.
**Table S2:** Differentially expressed miRNA relevant to prostate cancer according to the ultrasound phenotype.
**Table S3:** The AUC performance of the different methods and data sets split into the 10 runs.

## Data Availability

The data presented in this study are available on request from the corresponding authors (Q.Y. and X.L). The data are not publicly available owing to hospital regulations.
