# Supplementary material for: A New Radiotranscriptomic Approach to Analyze Combined Sets of T3b Stage‐Specific Genes and Radiomic Features in Prostate Cancer
Source: Cancer Rep (Hoboken). 2025 Dec 12;8(12):e70391. doi: 10.1002/cnr2.70391 (PMC12700717; doi:10.1002/cnr2.70391)
Supplement: Supplementary file 1 — Figure S1: Evaluation models of prostate cancer based on radiomics features. (A) LR = logistic regression mode. (B) SVM = support vector machine mode. (C) RF = random forest mode. Figure S2: Functional characteristics of the miRNAs specific to stage T2c in PCa. (A) The heat map for the top 51 differentially expressed (DE) miRNAs between T2c tumor samples and healthy samples. Blue denotes down‐regulation whereas red denotes up‐regulation. The dendrograms on the upper and left sides show the hierarchical clustering tree of samples and miRNAs, respectively. (B) A Venn diagram of the overlap between the DE miRNAs identified for T2c versus healthy samples, stage T3b versus healthy samples, and all tumor samples versus healthy samples. (C) Bubble chart shows the enriched functional terms specificity of the T2c miRNAs that are exclusively deregulated in the T2c tumor samples. Figure S3: Functional characteristics of genes specific to stage T3b in PCa. (A) The heat map for the top 710 differentially expressed genes (DEG) between T3b tumor samples and healthy samples. Blue denotes down‐regulation whereas red denotes up‐regulation. The dendrograms on the upper and left sides show the hierarchical clustering tree of samples and genes, respectively. (B) A Venn diagram of the overlap between the DEGs identified for T2c versus healthy samples, stage T3b versus healthy samples, and all tumor samples versus healthy samples. (C) Bubble chart shows the top 20 enriched Gene Ontology (GO) terms of biological processes of 710 genes that are exclusively deregulated in the T3b tumor samples, based on the GO semantic similarities. Color and size of the bubble represented enrichment significance and the number of DEGs enriched in GO terms, respectively. Q‐value < 0.05 was defined as significantly enriched. Table S1: Differentially expressed genes relevant to prostate cancer according to the ultrasound phenotype. Table S2: Differentially expressed miRNA relevant to prostate cancer according [file CNR2-8-e70391-s001.docx]

**Supplementary Files**

Decoding the Tumor Progression Phenotype of Prostate Cancer Using a Radiotranscriptomic Approach


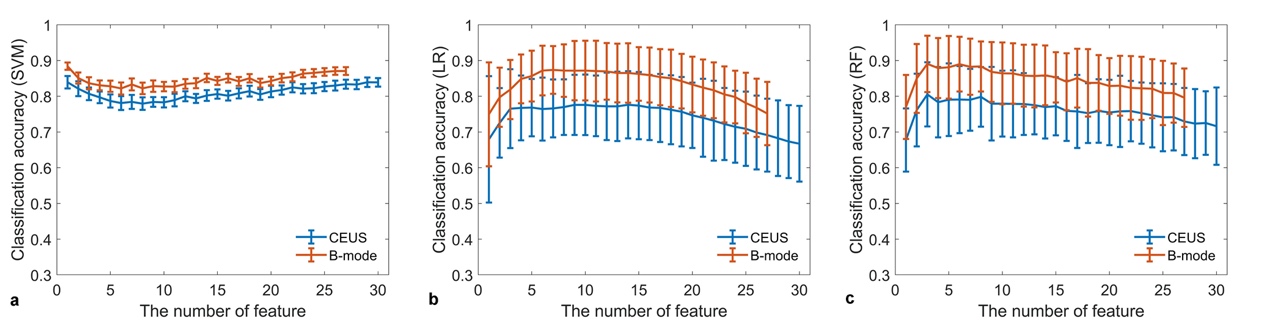


Figure S1. Evaluation models of prostate cancer based on radiomics features. (A) LR = logistic regression mode. (B) SVM = support vector machine mode. (C) RF = random forest mode.


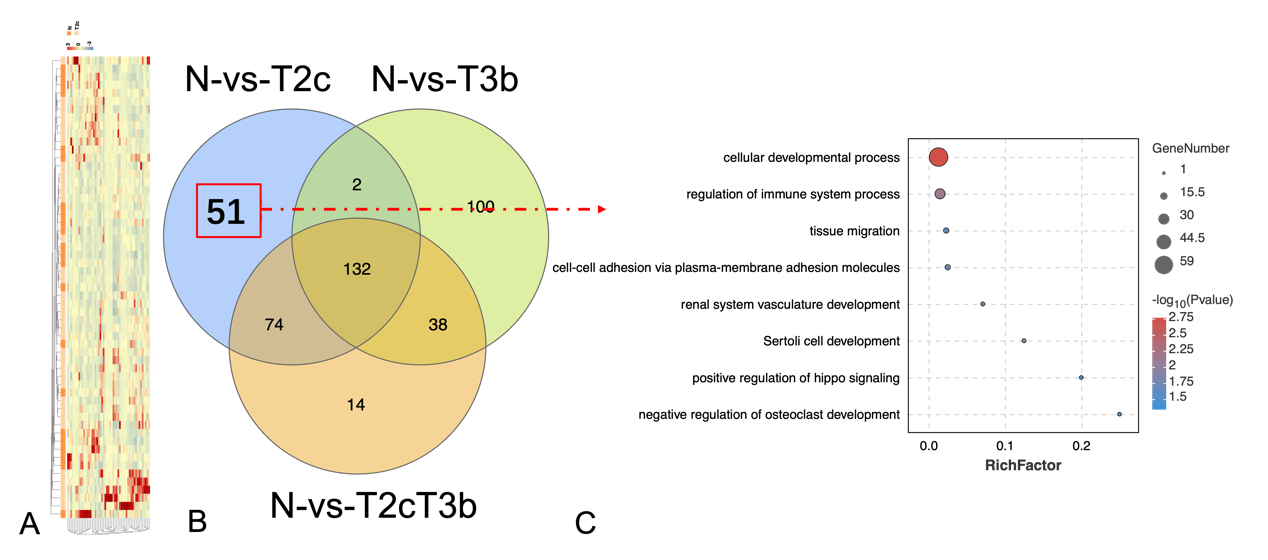


Figure S2. Functional characteristics of the miRNAs specific to stage T2c in PCa. (A) The heat map for the top 51 differentially expressed (DE) miRNAs between T2c tumor samples and healthy samples. Blue denotes down-regulation whereas red denotes up-regulation. The dendrograms on the upper and left sides show the hierarchical clustering tree of samples and miRNAs, respectively. (B) A Venn diagram of the overlap between the DE miRNAs identified for T2c versus healthy samples, stage T3b versus healthy samples, and all tumor samples versus healthy samples. (C) Bubble chart shows the enriched functional terms specificity of the T2c miRNAs that are exclusively deregulated in the T2c tumor samples.


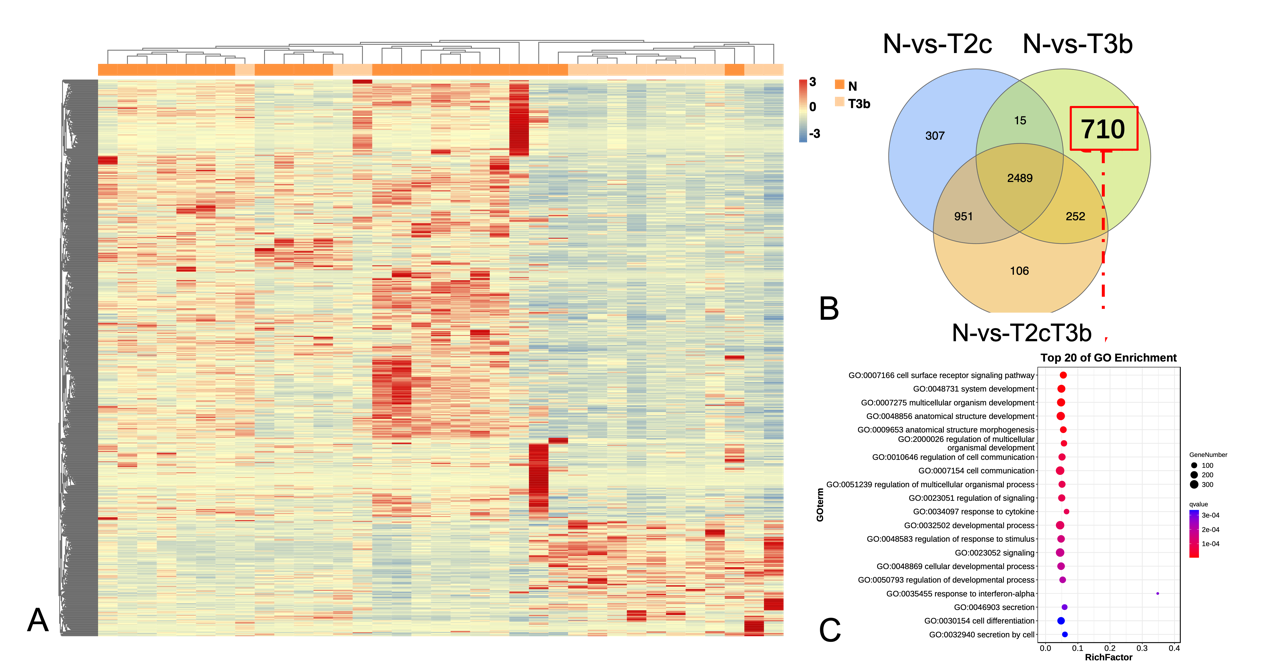


Figure S3. Functional characteristics of genes specific to stage T3b in PCa. (A) The heat map for the top 710 differentially expressed genes (DEG) between T3b tumor samples and healthy samples. Blue denotes down-regulation whereas red denotes up-regulation. The dendrograms on the upper and left sides show the hierarchical clustering tree of samples and genes, respectively. (B) A Venn diagram of the overlap between the DEGs identified for T2c versus healthy samples, stage T3b versus healthy samples, and all tumor samples versus healthy samples. (C) Bubble chart shows the top 20 enriched Gene Ontology (GO) terms of biological processes of 710 genes that are exclusively deregulated in the T3b tumor samples, based on the GO semantic similarities). Color and size of the bubble represented enrichment significance and the number of DEGs enriched in GO terms, respectively. Q-value < 0.05 was defined as significantly enriched.

**Table S1: Differentially expressed genes relevant to prostate cancer according to the ultrasound phenotype**

| **US Phenotype** | **T2 Stage**  **r** | **T2 Stage**  ***p*-value** | **T3 Stage**  **r** | **T3 Stage**  ***p*-value** |
| --- | --- | --- | --- | --- |
| **B-mode US** |  |  |  |  |
| **ca2-GLRLM-RLV** |  |  |  |  |
| RPL29 | -0.07 | 0.69 | 0.08 | 0.80 |
| RPS7 | 0.07 | 0.67 | 0.15 | 0.62 |
| FZD4 | -0.05 | 0.79 | 0.73 | *P*<0.01 |
| **cv2-GLSZM-LZHGE** |  |  |  |  |
| RPL29 | 0.04 | 0.84 | 0.63 | *P*<0.05 |
| RPS7 | -0.20 | 0.24 | 0.15 | *P*<0.01 |
| FZD4 | 0.17 | 0.33 | 0.89 | 0.62 |
| **ca2−GLSZM−LZE** |  |  |  |  |
| RPL29 | 0.03 | 0.86 | 0.47 | 0.10 |
| RPS7 | -0.19 | *P*<0.01 | 0.62 | 0.12 |
| FZD4 | -0.17 | 0.62 | -0.09 | 0.75 |
| **cv2−GLSZM−LZE** |  |  |  |  |
| RPL29 | 0.04 | 0.84 | 0.64 | *P*<0.05 |
| RPS7 | -0.19 | 0.26 | 0.89 | *P*<0.01 |
| FZD4 | -0.17 | 0.34 | 0.15 | 0.62 |
| **ch2−GLSZM−LZE** |  |  |  |  |
| RPL29 | 0.03 | 0.85 | 0.62 | *P*<0.05 |
| RPS7 | -0.19 | 0.26 | 0.87 | *P*<0.01 |
| FZD4 | -0.17 | 0.34 | 0.09 | 0.76 |
| **cv2−GLSZM−LZLGE** |  |  |  |  |
| RPL29 | 0.04 | 0.82 | -0.64 | 0.19 |
| RPS7 | -0.19 | 0.27 | 0.89 | *P*<0.01 |
| FZD4 | -0.17 | 0.34 | 0.15 | 0.62 |
| **CEUS** |  |  |  |  |
| **NGTDM−Busyness** |  |  |  |  |
| RPL29 | -0.02 | 0.90 | 0.60 | 0.30 |
| RPS7 | -0.20 | 0.24 | 0.65 | *P*<0.05 |
| FZD4 | 0.06 | 0.75 | -0.23 | 0.44 |
| **cd1−NGTDM−Coarseness** |  |  |  |  |
| RPL29 | 0.11 | 0.54 | 0.63 | 0.02 |
| RPS7 | 0.40 | *P*<0.05 | 0.83 | *P*<0.01 |
| FZD4 | 0.33 | 0.05 | 0.48 | 0.09 |
| **cv1−GLSZM−ZSV** |  |  |  |  |
| RPL29 | 0.17 | 0.33 | 0.64 | *P*<0.05 |
| RPS7 | 0.31 | 0.07 | 0.89 | *P*<0.01 |
| FZD4 | 0.41 | *P*<0.05 | 0.17 | 0.57 |
| **ch2−Global−Skewness** |  |  |  |  |
| RPL29 | -0.22 | 0.21 | 0.23 | 0.45 |
| RPS7 | 0.17 | 0.32 | 0.01 | 0.98 |
| FZD4 | 0.06 | 0.70 | 0.62 | *P*<0.05 |
| **ca2−GLCM−Correlation** |  |  |  |  |
| RPL29 | 0.21 | 0.24 | -0.07 | 0.81 |
| RPS7 | 0.24 | 0.15 | -0.35 | 0.23 |
| FZD4 | -0.06 | 0.70 | -0.77 | *P*<0.01 |
| **Microvascular perfusion** |  |  |  |  |
| **Beta^-1^** |  |  |  |  |
| RPL29 | -0.16 | 0.35 | 0.19 | 0.54 |
| RPS7 | -0.22 | 0.20 | -0.12 | 0.69 |
| FZD4 | -0.07 | 0.67 | -0.59 | *P*<0.05 |
| **RBF** |  |  |  |  |
| RPL29 | 0.03 | 0.89 | -0.67 | *P*<0.01 |
| RPS7 | -0.15 | 0.39 | -0.69 | *P*<0.01 |
| FZD4 | -0.03 | 0.86 | -0.28 | 0.36 |

**Table S2: Differentially expressed miRNA relevant to prostate cancer according to the ultrasound phenotype**

| **US Phenotype** | T2-Stage  r | T2-Stage  *P*-value | T3-Stage  r | T3-Stage  *P*-value |
| --- | --- | --- | --- | --- |
| **B-mode US** |  |  |  |  |
| **cv1−GLRLM−GLV** |  |  |  |  |
| has-miR-9-3p | -0.10 | 0.54 | 0.68 | *P*<0.05 |
| has-miR-6510-3p | 0.16 | 0.36 | -0.11 | 0.71 |
| has-miR-374c-5p | -0.04 | 0.82 | 0.60 | 0.29 |
| **ch2−GLSZM−LGZE** |  |  |  |  |
| has-miR-9-3p | -0.03 | 0.83 | 0.83 | *P*<0.01 |
| has-miR-6510-3p | 0.15 | 0.38 | 0.02 | 0.96 |
| has-miR-374c-5p | 0.02 | 0.93 | 0.67 | *P*<0.01 |
| **ch2−GLRLM−LGRE** |  |  |  |  |
| has-miR-9-3p | -0.04 | 0.82 | 0.81 | *P*<0.01 |
| has-miR-6510-3p | -0.14 | 0.44 | 0.02 | 0.94 |
| has-miR-374c-5p | -0.03 | 0.89 | 0.65 | *P*<0.05 |
| **ch1−Global−Variance** |  |  |  |  |
| has-miR-9-3p | 0.13 | 0.46 | 0.69 | *P*<0.01 |
| has-miR-6510-3p | -0.13 | 0.46 | -0.44 | 013 |
| has-miR-374c-5p | -0.12 | 0.49 | 0.62 | *P*<0.05 |
| **ch1−Global−Skewness** |  |  |  |  |
| has-miR-9-3p | 0.01 | 0.98 | -0.68 | *P*<0.05 |
| has-miR-6510-3p | 0.01 | 0.99 | 0.10 | 0.70 |
| has-miR-374c-5p | 0.08 | 0.64 | -0.51 | 0.07 |
| **ca2−NGTDM−Strength** |  |  |  |  |
| has-miR-9-3p | -0.03 | 0.88 | 0.67 | *P*<0.05 |
| has-miR-6510-3p | -0.01 | 0.95 | 0.06 | 0.84 |
| has-miR-374c-5p | -0.14 | 0.42 | 0.37 | 0.22 |
| **cv2−GLSZM−LZLGE** |  |  |  |  |
| has-miR-9-3p | -0.03 | 0.85 | -0.17 | 0.58 |
| has-miR-6510-3p | -0.09 | 0.58 | 0.70 | *P*<0.01 |
| has-miR-374c-5p | -0.11 | 0.51 | -0.18 | 0.17 |
| **cv2−GLSZM−LZHGE** |  |  |  |  |
| has-miR-9-3p | -0.03 | 0.86 | -0.17 | 0.58 |
| has-miR-6510-3p | -0.08 | 0.61 | 0.71 | *P*<0.01 |
| has-miR-374c-5p | -0.12 | 0.50 | -0.18 | 0.17 |
| **CEUS** |  |  |  |  |
| **ch1−Global−Skewness** |  |  |  |  |
| has-miR-9-3p | -0.03 | 0.82 | -0.60 | *P*<0.05 |
| has-miR-6510-3p | 0.09 | 0.59 | -0.23 | 0.44 |
| has-miR-374c-5p | 0.06 | 0.71 | -0.41 | 0.17 |
| **ca2−NGTDM−Strength** |  |  |  |  |
| has-miR-9-3p | 0.05 | 0.74 | 0.85 | *P*<0.01 |
| has-miR-6510-3p | -0.29 | 0.08 | -0.38 | 0.19 |
| has-miR-374c-5p | -0.18 | 0.30 | 0.85 | *P*<0.01 |
| **ca2−GLCM−Correlation** |  |  |  |  |
| has-miR-9-3p | 0.11 | 0.54 | 0.62 | *P*<0.05 |
| has-miR-6510-3p | -0.17 | 0.31 | -0.36 | 0.22 |
| has-miR-374c-5p | 0.06 | 0.75 | 0.61 | *P*<0.05 |
| **NGTDM−Busyness** |  |  |  |  |
| has-miR-9-3p | 0.08 | 0.64 | 0.15 | 0.61 |
| has-miR-6510-3p | 0.07 | 0.69 | 0.60 | 0.29 |
| has-miR-374c-5p | -0.14 | 0.43 | -0.02 | 0.94 |
| **cv1−GLSZM−ZSV** |  |  |  |  |
| has-miR-9-3p | -0.09 | 0.61 | -0.17 | 0.57 |
| has-miR-6510-3p | 0.16 | 0.35 | 0.76 | *P*<0.05 |
| has-miR-374c-5p | 0.22 | 0.21 | -0.22 | 0.48 |
| **ch1−GLRLM−RLV** |  |  |  |  |
| has-miR-9-3p | -0.07 | 0.67 | -0.23 | 0.43 |
| has-miR-6510-3p | 0.02 | 0.91 | 0.79 | *P*<0.01 |
| has-miR-374c-5p | -0.01 | 0.93 | -0.46 | 0.11 |
| **cd1−NGTDM−Coarseness** |  |  |  |  |
| has-miR-9-3p | -0.15 | 0.38 | -0.07 | 0.82 |
| has-miR-6510-3p | 0.11 | 0.53 | 0.64 | *P*<0.05 |
| has-miR-374c-5p | 0.23 | 0.18 | -0.62 | 0.61 |
| **Microvascular perfusion** |  |  |  |  |
| **RBV** |  |  |  |  |
| has-miR-9-3p | -0.06 | 0.74 | -0.56 | *P*<0.05 |
| has-miR-6510-3p | -0.01 | 0.96 | 0.06 | 0.85 |
| has-miR-374c-5p | 0.21 | 0.22 | -0.16 | *P*<0.05 |
| **Beta^-1^** |  |  |  |  |
| has-miR-9-3p | -0.13 | 0.44 | 0.62 | *P*<0.05 |
| has-miR-6510-3p | -0.15 | 0.40 | -0.53 | 0.06 |
| has-miR-374c-5p | -0.19 | 0.27 | 0.66 | *P*<0.05 |
| **RT** |  |  |  |  |
| has-miR-9-3p | -0.07 | 0.68 | -0.38 | 0.19 |
| has-miR-6510-3p | 0.10 | 0.54 | 0.63 | *P*<0.05 |
| has-miR-374c-5p | 0.21 | 0.22 | -0.46 | 0.11 |

**Table S3. The AUC performance of the different methods and data sets split into the 10 runs**

|  |  | **Random Forest** | **Naive Bayes** | **Support Vector Machines** |
| --- | --- | --- | --- | --- |
|  |  | **AUC** | **AUC** | **AUC** |
| **Clinical data** | 1 | 0.58 | 0.62 | 0.95 |
|  | 2 | 059 | 0.69 | 0.97 |
|  | 3 | 0.58 | 0.67 | 0.97 |
|  | 4 | 0.58 | 0.69 | 0.95 |
|  | 5 | 0.60 | 0.66 | 0.94 |
|  | 6 | 0.61 | 0.62 | 0.95 |
|  | 7 | 0.59 | 0.67 | 0.95 |
|  | 8 | 0.59 | 0.76 | 0.94 |
|  | 9 | 0.59 | 0.70 | 0.95 |
|  | 10 | 0.56 | 0.67 | 0.96 |
| **Average** | | 0.585 | 0.675 | 0.953 |
|  |  | **Random Forest** | **Naive Bayes** | **Support Vector Machines** |
|  |  | **AUC** | **AUC** | **AUC** |
| T**ranscriptomics** | 1 | 0.60 | 0.93 | 0.91 |
|  | 2 | 0.57 | 0.94 | 0.86 |
|  | 3 | 0.55 | 0.94 | 0.84 |
|  | 4 | 0.56 | 0.93 | 0.85 |
|  | 5 | 0.59 | 0.94 | 0.91 |
|  | 6 | 0.58 | 0.93 | 0.93 |
|  | 7 | 0.59 | 0.94 | 0.89 |
|  | 8 | 0.59 | 0.94 | 0.91 |
|  | 9 | 0.59 | 0.93 | 0.93 |
|  | 10 | 0.59 | 0.92 | 0.94 |
| **Average** | | 0.583 | 0.716 | 0.898 |
|  |  | **Random Forest** | **Naive Bayes** | **Support Vector Machines** |
|  |  | **AUC** | **AUC** | **AUC** |
| **Radiomics** | 1 | 0.90 | 0.99 | 0.99 |
|  | 2 | 0.93 | 0.97 | 0.99 |
|  | 3 | 0.93 | 0.99 | 0.99 |
|  | 4 | 0.91 | 0.99 | 0.1 |
|  | 5 | 0.93 | 0.99 | 0.99 |
|  | 6 | 0.92 | 0.99 | 0.97 |
|  | 7 | 0.91 | 0.93 | 0.99 |
|  | 8 | 0.93 | 0.85 | 0.98 |
|  | 9 | 0.92 | 0.91 | 0.99 |
|  | 10 | 0.93 | 0.96 | 0.98 |
| **Average** | | 0.921 | 0.957 | 0.998 |
|  |  | **Random Forest** | **Naive Bayes** | **Support Vector Machines** |
|  |  | **AUC** | **AUC** | **AUC** |
| **Combination** | 1 | 0.89 | 0.99 | 0.99 |
|  | 2 | 0.88 | 0.96 | 1 |
|  | 3 | 0.87 | 0.99 | 0.99 |
|  | 4 | 0.89 | 0.98 | 1 |
|  | 5 | 0.89 | 0.09 | 0.99 |
|  | 6 | 0.89 | 0.92 | 0.98 |
|  | 7 | 0.89 | 0.97 | 0.99 |
|  | 8 | 0.89 | 0.88 | 0.99 |
|  | 9 | 0.88 | 0.97 | 1 |
|  | 10 | 0.88 | 0.97 | 0.99 |
| **Average** | | 0.887 | 0.956 | 0.996 |
